# Association Between Risk for Prediabetes and Type 2 Diabetes Mellitus Prevention Among Faculty Members and Administrative Staff of a Saudi University

**DOI:** 10.7759/cureus.41926

**Published:** 2023-07-15

**Authors:** Abdullah I Al Shuwaysh, Eduardo L Fabella, Mohammed Al Hassan, Yasser Taher Al Hassan, Abdullah Al Hassan, Hussam Al Majed, Ahmad Al Nahwi, Hassan Al Howayshel, Ali Al Abdi

**Affiliations:** 1 Department of Public Health, College of Applied Medical Sciences, King Faisal University, Al-Ahsa, SAU; 2 Health Programs Department, Public Health Directorate, Al-Ahsa Directorate for Health Affairs, Ministry of Health, Al-Ahsa, SAU

**Keywords:** university faculty and staff, prediabetes risk, type 2 diabetes mellitus risk, type 2 diabetes mellitus prevention, prediabetes

## Abstract

Background: Diabetes mellitus prevalence continues to rise globally, causing disability and decreased productivity among patients, a significant strain on healthcare systems, and a burden on national economies. In 2021, diabetes will affect approximately 537 million adults. The rising prevalence of prediabetes worldwide also poses a significant public health threat, as it is estimated that by 2030, more than 470 million individuals will be prediabetic.

Objective: This study aimed to determine the association between the risk of prediabetes and the level of Type 2 Diabetes Mellitus (T2DM) prevention among faculty members and administrative staff of a Saudi university.

Methods: An analytic cross-sectional study design was utilized. The prediabetes risk of respondents was assessed using a risk test developed by the CDC, while the participants’ diabetes prevention practices were determined using a researcher-developed questionnaire. Data were collected from 360 selected faculty members and administrative staff of three randomly selected health colleges and three non-health colleges at King Faisal University, Hofuf, Al-Ahsa, Saudi Arabia, between September 25 and October 13, 2022. The collected data were subjected to estimation of proportion and logistic regression analyses using Epi Info^TM^ version 7.

Results: Nearly 40% of respondents (39.72%, 95% CI: 34.80, 44.86) were found to be at high risk for prediabetes. The majority of university faculty and administrative staff consistently practiced T2DM preventive measures related to the limitation of processed food consumption, smoking cessation, and regular checking of weight and the nutritional value of food. However, there was poor T2DM prevention practice in terms of exercise, consumption of sweetened beverages, and stress reduction. Those who had a high prediabetes risk were 1.17 times more likely to engage in T2DM prevention practices. However, they were found to be 19% less likely to perform T2DM prevention practices when sociodemographic variables were held constant.

Conclusion: Prediabetes risk was prevalent among Saudi university faculty and administrative staff. T2DM prevention was not consistently practiced by those who had a high risk for prediabetes. High prediabetes risk was negatively associated with the level of T2DM prevention.

## Introduction

The prevalence of diabetes mellitus continues to rise globally. The disease poses a significant threat to the health and well-being of not only the individuals affected but their families and societies as well [1]. Diabetes mellitus is a chronic, non-communicable disease characterized by elevated levels of blood glucose, which leads to significant damage to the cardiovascular system, the eyes, kidneys, and nerves over time [2].

Nearly all parts of the world have seen significant increases in the prevalence of diabetes in recent decades. Today, it is estimated that 415 million people worldwide have the disease [3]. Genetic influences, a sedentary lifestyle, and the spread of Westernized eating habits over the past several years have resulted in the rise of non-communicable diseases such as T2DM. The increasing prevalence of diabetes is a global phenomenon [4].

The most recent data from the International Diabetes Federation (IDF) indicates that diabetes mellitus affects approximately 537 million adults between the ages of 20 and 79 globally. This number is projected to rise to 643 million by 2030 and 783 million by 2045. It is estimated that close to 240 million persons with diabetes mellitus are undiagnosed. Furthermore, in 2021, the disease caused 6.7 million deaths, and 541 million adults worldwide are at increased risk of developing Type 2 diabetes mellitus [1].

Diabetes mellitus causes significant financial losses to patients, their families, and healthcare systems, including decreased productivity and a burden on national economies [5]. Because of its chronic nature and related consequences, diabetes is considered a costly disease [6]. In 2021, the IDF stated that diabetes mellitus caused at least USD 966 billion in health expenditures, or 9% of total spending on adults globally [7].

A study conducted in 2017 in the US by the American Diabetes Association (ADA) showed that 30% of the total medical expenditures in the US are spent on hospital inpatient care because of diabetes. An additional 30% was used for drug prescriptions for the management of diabetic complications, while antidiabetic agents and diabetes supplies represent 15% of total medical expenditures in the country [5].

Diabetes has reached epidemic levels, threatening the economy and well-being of individuals globally and nationally [7]. Saudi Arabia is not an exception to this pandemic [8]. Saudi Arabia ranks second among Middle Eastern countries in terms of diabetes mellitus prevalence [9]. Over the next 20 years, it is anticipated that the prevalence of diabetes will rise in Saudi Arabia, similar to global trends in other countries [10]. The International Diabetes Federation reported that Saudi Arabia is one of the top 10 countries with the highest prevalence of diabetes mellitus among adults aged 20 to 79 [11].

According to Alghamdi et al., 3,852,000 cases of diabetes were reported in Saudi Arabia in 2017. It has been reported that every year in Saudi Arabia, 5,000-foot amputations are performed due to diabetes. Moreover, around 23,420 annual deaths are attributable to the disease [12]. The overall health expenditure of Saudi Arabia in 2014 was 180 billion Saudi Riyals, of which 25 billion (or 13.9% of the total) were spent on managing diabetes mellitus for the entire Saudi diabetic population [13].

A 2016 Saudi study found that more than 50% of people aged 30 and older had either diabetes (25.4%) or pre-diabetes (25.5%), with 40.3% of diabetic patients being completely unaware that they had the disease [10]. The IDF has estimated the prevalence of diabetes in KSA in 2030 at 20.4% and 21.4% in 2045. [14].

Prediabetes is a major health condition characterized by elevated blood sugar but not high enough to be diagnosed as Type 2 diabetes mellitus [15]. The worldwide prevalence of prediabetes is rising, and it is estimated that by 2030, more than 470 million individuals will be prediabetic [16]. In Saudi Arabia, 25.5% of individuals aged 30 or younger have prediabetes, putting more than three million people at risk of developing diabetes mellitus [17].

Controlling prediabetes can greatly decrease or stop the progression to T2DM and avoid consequences like myocardial infarction and stroke. To stop prediabetes progression to T2DM, prediabetes screening should be prioritized for people with T2DM risk factors. [18]. The purpose of prediabetes screening is to detect and treat this issue sooner rather than later to prevent the progression of the disease and its adverse effects, such as heart attack, renal failure, impaired vision, and even death [19].

Prediabetes is reversible. Simple lifestyle changes have been proven to stop or delay the progression of prediabetes into Type 2 diabetes mellitus [20]. According to a study that was conducted in 2016 in Riyadh, Saudi Arabia, the alarming increase in diabetes to over 25% of the adult population has been caused by unhealthy dietary habits and a decline in physical activity across the Kingdom of Saudi Arabia [10]. In the southwest region of Saudi Arabia, 58.3% of adults were not adhering to the international physical activity recommendations [21].

Effective lifestyle change is the suggested first-line management strategy for prediabetes. Reducing and modifying caloric intake to achieve weight loss in overweight or obese people, quitting tobacco products, engaging in moderate physical activities, getting enough sleep, and reducing stress are among the lifestyle changes that have been recommended to address prediabetes [18]. It has been demonstrated in various studies that lifestyle modification among prediabetics can effectively prevent progression to T2DM [16].

Despite the significant prevalence of Type 2 diabetes mellitus in Saudi Arabia, there is a scarcity of studies on prediabetes and Type 2 diabetes prevention practices in the Kingdom. Literature searches in the major research databases yielded limited published studies regarding the association between risk for prediabetes and Type 2 diabetes prevention practices globally and locally. While several studies were conducted recently to determine diabetes mellitus prevalence in Saudi populations, only a few studies have been carried out regarding prediabetes and even fewer related to prediabetes risk and T2DM prevention. Thus, this study was conducted to determine the prediabetes risk and its association with T2DM prevention practices.

## Materials and methods

A cross-sectional study was conducted among faculty members and administrative staff of King Faisal University, a government university located in Hofuf, Al-Ahsa, in the Eastern Region of Saudi Arabia. King Faisal University consists of 11 non-health colleges and four health colleges.

A multistage sampling procedure was utilized. Three health colleges and three non-health colleges were selected by simple random sampling at the first stage of the sampling procedure. This was followed by stratified sampling to identify faculty members and administrative staff from whom data was collected. All faculty and administrative staff were deemed eligible to participate in the survey regardless of their sex, age, employment classification, employment duration at the university, and their total years of work experience.

The minimum sample size was computed using OpenEpi (https://www.openepi.com/) to be 310 using the following parameters: population of university faculty and administrative staff of 3,585, expected frequency of 18.5% based on diabetes prevalence in Saudi Arabia as reported by the IDF in 2017 [22], confidence level of 90%, allowable error of 10%, and a design effect of two. A total of 360 teaching and administrative staff responded and consented to participate in the study from the following colleges: the College of Applied Medical Sciences, the College of Medicine, the College of Clinical Pharmacy, the College of Business Administration, the College of Science, and the College of Veterinary Medicine.

The prediabetes risk of respondents was assessed using the CDC Prediabetes Risk Test. The Prediabetes Risk Test was adapted from the CDC [23] and includes questions related to age, history of gestational diabetes for women, family history of diabetes, personal history of elevated blood pressure, race, whether a person is physically active or not, as well as weight and height. The CDC Prediabetes Risk Test considers a person with a score of five or higher at risk for prediabetes, while those who scored otherwise were considered at low risk.

The researcher-developed questionnaire on T2DM prevention was used to determine the level of T2DM prevention practices. All questions in this section were based on the recommendations of the US CDC for T2DM Prevention. The tool was validated by health professionals at the university and pre-tested independently among people of similar characteristics as the study population. The T2DM prevention practices questionnaire consisted of questions related to exercise, processed food consumption, sweetened beverage consumption, smoking cessation, stress reduction, weight maintenance, and checking the nutritional value of food. Responses were used to evaluate the participants’ level of compliance with recommended T2DM preventive practices. A respondent whose score was at or above the mean of the prevention practices was categorized as having “high-level prevention,” while those who scored otherwise were regarded as having “low-level prevention”.

The status of whether a person was practicing T2DM prevention was based on their response to questions related to T2DM prevention practices, such that a “yes” response was counted as one score of prevention. For exercise, the score was based on the participants' answers to the question. “How often do you engage in the following physical activities”. A response of “never” or “once a week” was assigned a value of 0, while a response of “always” or “2-3 times per week” was assigned a value of 1. For ultra-processed food and sweetened beverage consumption, the score was based on the participants’ responses to the question “How often do you consume the following?” A response of “never” or “once a week” was assigned a value of 1 while a response of “always” or “2-3 times per week” was assigned a value of 0.

Data were exported from Google Forms (California, USA) to Microsoft Excel (Redmond, USA) and were subsequently cleaned for missing information before statistical analysis using Epi Info^TM^ version 7 software. Descriptive analysis was applied to the demographic data. An estimation of the proportion of faculty and administrative staff who were at risk for prediabetes was performed. Additionally, estimation of the proportion was applied to determine the proportion of university faculty and administrative staff who practice T2DM prevention practices. A simple logistic regression was performed to determine the crude association between the university faculty members and administrative staff’s risk for prediabetes and their Type 2 diabetes prevention practices. Finally, multiple logistic regression was performed to account for the possible effects of confounders.

Data was collected from respondents using the self-administered questionnaire distributed by Google Forms from September 25 to October 13, 2022. Respondents participated on a voluntary basis and were assured of their anonymity and the confidentiality of their responses. Ethical clearance was obtained from the Research Ethics Committee of the Deanship of Scientific Research, King Faisal University, Hofuf, Al-Ahsa, Kingdom of Saudi Arabia (KFU - REC/2022 - 9 - 17 - ETHICS168).

## Results

Demographic characteristics of university faculty members and administrative staff

The demographic characteristics of university faculty members and administrative staff who participated in the study are shown in Table 1.

**Table 1:** Demographic characteristics of university faculty members and administrative staff (n=360)

Variable	Frequency	%
Age		
Less than 40	153	42.50
40-49	147	40.83
50-59	52	14.44
60 and higher	8	2.22
Sex		
Male	210	58.33
Female	150	41.67
Nationality		
Saudi	195	54.17
Non-Saudi	165	45.83
Job Classification		
Faculty	224	62.22
Administrative staff	136	37.78
College type		
Health	152	42.22
Non-health	208	57.78

Among the 360 respondents, majority (83.33%) were from the “less than 40” and the “40-49” age groups. There was a higher proportion of the faculty and administrative staff who were male (58.33%) than those who were female (41.67%). In terms of nationality, 54.17% were Saudis, while 45.83% were non-Saudis. Furthermore, 62.22% of all participants were teaching staff (faculty), while 37.78% were administrative staff. Lastly, the proportions of respondents from non-health colleges and health colleges were 57.78% and 42.22%, respectively.

Prediabetes risk among university faculty members and administrative staff

Faculty members and administrative staff who were at high risk for prediabetes comprised 39.72% (95%, CI: 34.80 to 44.86%) of the participants, while more than half (60.28%) of the respondents (95%, CI: 55.14 to 65.20%) were at low risk of being prediabetic.

The prediabetes risk of faculty members and administrative staff stratified by age, sex, nationality, job classification, and college type are depicted in Table 2.

**Table 2:** Distribution of university faculty members and administrative staff based on prediabetes risk (n=360)

Demographic Variable	High Risk			Low Risk		
	Count	%	95% CI	Count	%	95% CI
Age						
Less than 40	17	11.11	6.61 - 17.19	136	88.89	82.81 - 93.39
40-49	75	51.02	42.65 - 59.35	72	48.98	40.65 - 57.35
50-59	44	84.62	71.92 - 93.12	8	15.38	6.88 - 28.08
60 and higher	7	87.50	47.35 - 99.68	1	12.50	0.32 - 52.65
Sex						
Male	116	55.24	48.24 - 62.08	94	44.76	37.92 - 51.76
Female	27	18.00	12.21 - 25.10	123	82.00	74.90 - 87.79
Nationality						
Saudi	61	31.28	24.85 - 38.30	134	68.72	61.70 - 75.15
Non-Saudi	82	49.70	41.83 - 57.57	83	50.30	42.43 - 58.17
Job Classification						
Faculty	102	45.54	38.39 - 52.30	122	54.46	47.70 - 61.11
Administrative staff	41	30.15	22.58 - 38.60	95	69.85	61.40 - 77.42
College type						
Health	51	33.55	26.11 - 41.65	101	66.45	58.35 - 73.89
Non-health	92	44.23	37.37 - 51.26	116	55.77	48.74 - 62.63

Nearly nine out of every 10 (88.89%, 95% CI: 82.81 to 93.39%) of university staff who were less than 40 years old were categorized as having low prediabetes risk, while only one out of every 10 were at high risk. When grouped by age, there was a near equal proportion of respondents who were at high and low risk for prediabetes in the “40 to 49” age group. A high proportion of respondents with high prediabetes risk were found in the age groups “50 to 59” and “60 and above”.

When grouped by sex, a higher proportion of males were classified as having a high prediabetes risk. On the other hand, most females (82.0%) were classified as having low risk.

When participants were grouped by nationality, almost 70% of the Saudi respondents were classified as having a low risk of prediabetes, while a minority had a high risk. However, among the non-Saudis, the proportions of those who had high and low prediabetes risks were almost equal.

As for job classification, slightly more than half of respondents among the faculty (54.46%) were at low risk for prediabetes. Among the administrative staff, nearly seven out of 10 were at low prediabetes risk, while only three out of every 10 were categorized as having high risk.

A lower proportion of faculty and administrative staff from the health colleges were categorized as having high prediabetes risk. Similarly, among those from the non-health colleges, most of the respondents were classified as having low risk for prediabetes, while only four out of every 10 respondents were grouped as having high risk (44.23%).

T2DM prevention practices of university faculty members and administrative staff

**Figure 1:**
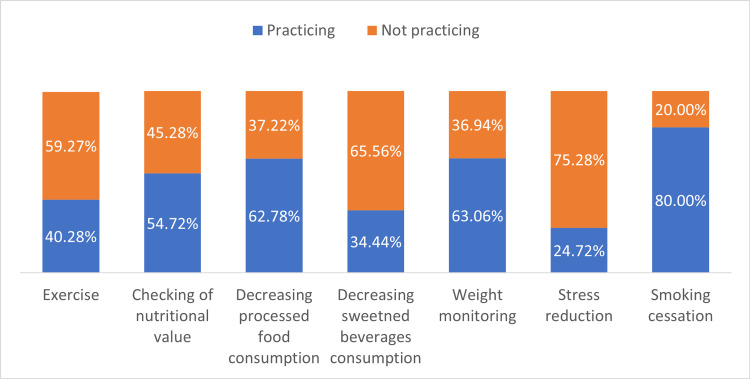
T2DM practices of university faculty and administrative staff

Figure 1 shows the distribution of university faculty members and administrative staff according to T2DM prevention practices.

The US CDC prescribes the following as essential components of T2DM prevention: exercise, nutritional value checking, reduction of processed food and sweetened beverage consumption, regular weight checks, reduction of stress, and smoking cessation.

In terms of exercise, four out of every 10 (40.28%, 95% CI: 35.34% to 45.42%) faculty members and administrative staff reported exercising regularly, while a higher proportion (59.27%, 95% CI: 54.58% to 64.66%) were classified as not exercising regularly. Walking was the most reported form of physical activity by the study participants, with 88.97% claiming to engage in this activity regularly. Weightlifting was the second most frequently practiced form of physical activity (67.59%), followed by jogging (57.24%), cycling (44.14%), and playing football (26.90%).

Checking the nutritional value of food and beverages was reported to be regularly done by more than half (54.72%, 95% CI: 49.56% to 59.79%) of the respondents.

Nearly six out of every 10 (62.78%, 95% CI: 57.67% to 67.61%) respondents declared not consuming processed food frequently to reduce their risk for developing T2DM, while nearly four out of every 10 faculty members and administrative staff reported consuming processed food “always” or “2-3 times per week”. Among the ultra-processed food products, hot dogs and instant noodles were the most frequently consumed (82.50%). Moreover, chips were consumed by 81.39% of all participants. Nearly eight out of every 10 (78.89%) respondents reported consuming ice cream, while a proportion of 70.28% consumed frozen hamburgers. Lastly, 40.28% of all respondents claimed to consume packaged bread.

As for sweetened beverages, 34.44% (95%, CI: 29.72% to 39.49%) of all participants stated that they avoid consuming sweetened beverages regularly. On the other hand, 65.56% (95%, CI: 60.51% to 70.28%) reported regular intake of sugary beverages. The types of sweetened beverages consumed varied. More than half (62.50%) of respondents declared consuming carbonated beverages. Sweetened tea was consumed by 46.11% of all respondents. The consumption of sweetened coffee and sweetened fruit juices was 55.44% and 59.44%, respectively.

In the matter of regular weight checking, 63.06% (95% CI: 57.96% to 67.88%) of the respondents claimed to perform regular checking of their weight for T2DM prevention purposes, while more than a quarter of participants (36.94%, 95% CI: 32.12% to 42.04%) do not.

Stress reduction was practiced by a minority (24.72%, 95% CI: 20.55% to 29.43%) of faculty and administrative staff, while the majority (75.28%, 95% CI: 70.57% to 79.45%) stated not practicing any forms of stress reduction.

Lastly, there was a higher proportion of faculty and administrative staff who practiced smoking cessation as a form of T2DM prevention compared to those who did not practice smoking cessation: 80.00% (95%, CI: 75.56% to 83.81%) and 20.00% (95%, CI: 16.19% to 24.44%), respectively.

The distribution of university faculty members and administrative staff based on T2DM prevention practices stratified by the respondents’ sociodemographic variables is depicted in Table 3.

**Table 3:** Distribution of university faculty members and administrative staff based on T2DM prevention practices (n=360)

Demographic Variable	Low level of prevention			High level of prevention		
	Count	%	95% CI	Count	%	95% CI
Age						
Less than 40	76	49.67	41.50 - 57.86	77	50.33	42.14 - 58.5
40-49	81	55.10	46.69 - 63.31	66	44.90	36.69 - 53.31
50-59	27	51.92	37.63 - 65.99	25	48.08	34.01 - 62.37
60 and higher	6	75.00	34.91 - 96.81	2	25.00	3.19 - 65.09
Sex						
Male	126	60.00	53.03 - 66.68	84	40.00	33.32 - 46.97
Female	64	42.67	34.64 - 50.99	86	57.33	49.01 - 65.36
Nationality						
Saudi	101	51.79	44.54 - 58.99	94	48.21	41.01 - 55.46
Non-Saudi	89	53.94	46.02 - 61.72	76	46.06	38.28 - 53.98
Job Classification						
Faculty	125	55.80	49.04 - 62.41	99	44.20	37.59 - 50.96
Administrative staff	65	47.79	39.16 - 56.52	71	52.21	43.48 - 60.84
College type						
Health	75	49.34	41.15 – 57.56	77	50.66	42.44 - 58.85
Non-health	115	55.29	48.26 - 61.17	93	44.71	37.83 - 51.74

An almost equal proportion of respondents were categorized as having high-level and low-level T2DM prevention among those aged “less than 40”, “40-49”, and “50-59”. Among the age groups, the “60 and older” group had the highest proportion in the high-level prevention category (75.00%, 95% CI: 41.50% to 57.86%), while a minority were in the category of low-level prevention among respondents who were 60 or older.

When classified by sex, more than half of male participants (60.00%, 95% CI: 53.03% to 66.68%) were categorized as having a high level of T2DM prevention. However, most females (57.33%) were grouped as having a low level of prevention.

There was a near-equal proportion of Saudi participants who had high and low levels of T2DM prevention. However, among non-Saudis, slightly more than half (53.94%) were grouped as having a high level of prevention, whereas the remainder were classified as having a low level of T2DM prevention.

As regards job classification, a higher proportion of the faculty members were regarded as having a high level of T2DM prevention than those with a low level of prevention. Describing the administrative staff, nearly 50% (47.79, 95% CI: 39.16% to 56.52%) had a high level of T2DM prevention, while more than half of the respondents in this category had a low level of prevention.

An almost equal proportion of respondents were seen as having high-level and low-level T2DM prevention when the study participants were stratified according to college type. Furthermore, five out of every 10 of those from the non-health colleges were classified as having a high level of prevention, whereas only four out of every 10 respondents had a low level of prevention.

Association between risk for prediabetes and T2DM prevention

Upon application of simple logistic regression, prediabetes risk was found to be associated with the level of T2DM prevention. University faculty members and administrative staff who had a high prediabetes risk were 1.17 times more likely to practice T2DM prevention when compared to those with a low prediabetes risk. However, when age, sex, nationality, job classification, and type of college were held constant, those who had a high prediabetes risk were found to be 19% less likely to engage in T2DM prevention practices (Table 4).

**Table 4:** Association between risk for prediabetes and T2DM prevention practices among university faculty members and administrative staff

Variables	Crude OR (95% CI)	p-value	Adjusted OR (95% CI)	p-value
Risk				
High	1.1785 (0.7715 – 1.8002)	0.4474	0.8071 (0.4759 - 1.3688)	0.4266
Low	1.0000		1.0000	
Age				
High	1.2419 (0.8169 -1.8879)	0.3107	1.1214 (0.6711 - 1.8739)	0.6618
Low	1.0000		1.0000	
Sex				
Male	2.0156 (1.3173 - 3.0841)	0.0012	2.0676 (1.2978 - 3.2939)	0.0022
Female	1.0000		1.0000	
Nationality				
Saudi	0.9176 (0.6056 -1.3902)	0.6848	1.2249 (0.702 -2.1375)	0.4751
Non-Saudi	1.0000		1.0000	
Job Classification				
Faculty	0.7251 (0.4729 - 1.1118)	0.1405	0.682 (0.3941 - 1.1804)	0.1715
Administrative staff	1.0000		1.0000	
College type				
Health	0.7877 (0.517 -1.1981)	0.2647	0.7869 (0.507 -1.2214)	0.2853
Non-health	1.0000		1.0000	

The sociodemographic variables were independently associated with the level of T2DM prevention. Those whose age was 40 years old or older were 1.24 times more likely to have a high level of T2DM prevention compared to those who were less than 40 years of age. Furthermore, there was a significant association between sex and T2DM prevention practices. Compared to females, males were twice as likely to have a high level of T2DM prevention (p=0.0012). When the respondents were grouped by nationality, Saudis were 9% less likely to have a high level of T2DM prevention. As for job classification, the university faculty were 27% less likely to have a high level of T2DM prevention than the administrative staff. As for the college type, the faculty and administrative staff from health colleges were 21% less likely to have a high level of T2DM prevention when compared to the faculty and administrative staff from non-health colleges.

There were no significant associations between prediabetes risk, age, nationality, job classification, college type, and T2DM prevention practices when logistic regression was performed. However, a significant association was found between sex and T2DM prevention (p = 0.0022).

## Discussion

The study was conducted to determine the proportion of university faculty members and administrative staff who were at risk for prediabetes. Additionally, it sought to determine the proportion of university faculty members and administrative staff who engaged in T2DM prevention and whether an increased prediabetes risk was associated with the practice of T2DM prevention.

The findings of the study revealed that nearly four out of every 10 university staff were at high risk for prediabetes. A higher proportion of prediabetes risk was observed among male respondents. While there was a nearly equal proportion of respondents aged “less than 40”, “40-49, and “50-59” who were practicing T2DM prevention and those who were not practicing T2DM prevention, a higher proportion of respondents aged “60 and above” engaged in T2DM preventive practices.

Using the CDC Prediabetes Risk Test, the overall prevalence of high prediabetes risk in the current study was 39.72%. This result was almost similar to the findings of a study conducted among adults in Nepal concerning diabetes and prediabetes, in which 46.09% of all adults screened were classified as at high risk for DM [24]. Additionally, the results of the current study were consistent with the findings of an Indian community-based screening study targeting adults, in which 37% of all respondents were grouped as being at high risk for DM [25]. A 2021 Kuwaiti study has shown that the prevalence of prediabetes was estimated to be 47.9% by fasting blood glucose and 31.0% by HbA1c [26].

T2DM prevention practices recommended by the CDC were not consistently practiced by university faculty members and administrative staff. In the present study, physical inactivity was prevalent (59.27%). These results were comparable to a 2022 study conducted among T2DM patients in Makkah, in which it was stated that more than half of the population (51.6%) included in the study had low physical activity levels [27].

The most frequently practiced form of physical activity among respondents in the current study was walking, where nearly 90% of the study participants reported practicing such exercise always or 2-3 times per week. Similarly, according to a study in the United Arab Emirates by Al-Kaabi et al. involving T2DM patients, 78% of all participants claimed to practice walking as a form of physical activity [27,28]. Less than half of the respondents in the current study were regarded as physically active yet had a high prediabetes risk of 37.93%. A similar result was found in a 2019 Vietnamese study, where adults with T2DM were found to be less physically active [29].

Understanding and effectively implementing dietary habits is essential to reducing the risk of developing diabetes. A study on the relationship between ultra-processed food (UPF) consumption and T2DM reported that high consumption of ultra-processed food could increase the risk of developing T2DM in adult individuals and gestational diabetes in women [30]. In the current study, 37.22% of respondents claimed to consume processed food frequently. It was reported in a UK study targeting adults that participants consumed, on average, 1,823 kcal/day, and 54.3% of all those calories came from ultra-processed food consumption [31].

In the current study, the consumption of sweetened beverages was highly prevalent among respondents, where the majority (65.56%) reported that they consume sweetened beverages either always or 2-3 times per week. A 2022 Saudi study has also shown that 71% of all participants consumed sweetened beverages at least once a week [32]. Moreover, in the current study, 62.50% of the respondents reported consuming soda, while about six out of every 10 (59.44%) declared consuming sweetened fruit juices. In comparison, a study by AlFaris et al. showed that the consumption of soda and fruit drinks was 81.9% and 58.1%, respectively [33]. 

Both passive and active exposure to cigarette smoke have been demonstrated to increase the incidence of T2DM [34]. In the current study, 80% of the faculty and administrative staff were practicing smoking cessation as a T2DM prevention strategy.

It has been proposed that stress increases an individual’s risk of developing T2DM [35]. It was shown in a cohort 2014 study by Virtanen et al. that among participants with prediabetes and a high-risk score, 40.9% of those with psychological distress developed diabetes during the follow-up period, compared with 28.5% of those without distress [36]. In the present study, only 24.72% of the faculty and administrative staff engaged in stress reduction to decrease T2DM risk.

Studies have shown a strong correlation between weight and T2DM, with obese people having the highest risk of getting the disease [37]. In the current study, only 63.06% reported checking their weight regularly to avoid developing T2DM.

For individuals who are overweight or obese, weight reduction is an efficient way to prevent and enhance the management of T2DM. Diet is essential to weight loss, along with other lifestyle elements like exercise and behavior modification [18].

Lifestyle change is the suggested first-line management strategy for prediabetes. Reduction and modification of caloric intake to achieve weight loss in overweight or obese people, abstaining from tobacco products, engaging in moderate amounts of prescribed physical activity, getting enough sleep of the right kind and quantity, and reducing stress are all examples of lifestyle changes [18].

A greater risk of Type 2 diabetes exists in patients with prediabetes. Intensive lifestyle changes that prioritize exercise and weight loss are key strategies for postponing T2DM [38]. An 89% decrease in the incidence of diabetes is observed in high-risk individuals for T2DM who maintain their weight loss over time [39].

The study had several limitations. The results apply to the faculty and administrative staff of this university and do not necessarily reflect the association between the risk of prediabetes and the T2DM prevention practices of other university staff in other regions of Saudi Arabia or of the Saudi Arabian population in general. Practices related to T2DM are time-varying variables; therefore, respondents’ practices may not be truly reflected in the current study as the frequency of engaging in such practices may differ with time. The possibility of misclassification concerning T2DM practices may have occurred since responses to the survey were collected through a self-reported questionnaire, and no verification of individual responses was made.

## Conclusions

High prediabetes risk was prevalent among university faculty and administrative staff, especially among males. T2DM prevention was not consistently practiced by those at high risk for prediabetes. The study showed that there was satisfactory T2DM prevention in terms of decreasing ultra-processed food consumption, smoking cessation, and regular checking of weight and checking of the nutritional value of food. However, there was poor T2DM prevention in terms of exercise, reduction of consumption of sweetened beverages, and stress reduction. Finally, prediabetes risk was found to be associated with T2DM prevention. Faculty and administrative staff who are at risk for prediabetes were less likely to engage in T2DM prevention practices.

Recommendations

Saudi university faculty members and administrative staff, especially those aged 40 and older, will benefit from intensified educational programs for T2DM prevention, with greater emphasis on exercise, checking the nutritional value of food, reducing the consumption of sweetened beverages, and reducing stress. Advocacy for policy development regarding the possibility of increased taxation on sweetened beverages must be prioritized. Policies to create healthy and conducive environments to increase faculty members' and administrative staff’s engagement in physical activity are very much needed in Saudi universities. Finally, targeted screening programs concerning prediabetes through secondary prevention using fasting blood glucose, random blood sugar, and oral glucose tolerance tests should be regularly conducted in the university in collaboration with the university clinic, especially among faculty members and administrative staff at the highest risk for progression to diabetes.

## References

[1] (2022). IDF: Diabetes atlas 2022 reports. https://www.diabetesatlas.org.

[2] (2022). WHO: Diabetes. https://www.who.int/health-topics/diabetes.

[3] Harding JL, Pavkov ME, Magliano DJ, Shaw JE, Gregg EW (2019). Global trends in diabetes complications: a review of current evidence. Diabetologia.

[4] Kalan Farmanfarma KH, Ansari-Moghaddam A, Zareban I, Adineh HA (2020). Prevalence of type 2 diabetes in Middle-East: Systematic review and meta-analysis. Prim Care Diabetes.

[5] American Diabetes Association (2018). Economic costs of diabetes in the U.S. in 2017. Diabetes Care.

[6] Afroz A, Alramadan MJ, Hossain MN, Romero L, Alam K, Magliano DJ, Billah B (2018). Cost-of-illness of type 2 diabetes mellitus in low and lower-middle income countries: a systematic review. BMC Health Serv Res.

[7] Alshayban D, Joseph R (2020). Health-related quality of life among patients with type 2 diabetes mellitus in Eastern Province, Saudi Arabia: A cross-sectional study. PLoS One.

[8] Alotaibi A, Perry L, Gholizadeh L, Al-Ganmi A (2017). Incidence and prevalence rates of diabetes mellitus in Saudi Arabia: An overview. J Epidemiol Glob Health.

[9] AlQuaiz AM, Alrasheed AA, Kazi A, Batais MA, Alhabeeb KM, Jamal A, Fouda MA (2021). Is 25-hydroxyvitamin D associated with glycosylated hemoglobin in patients with type 2 diabetes mellitus in Saudi Arabia? A population based study. Int J Environ Res Public Health.

[10] Al Dawish MA, Robert AA, Braham R, Al Hayek AA, Al Saeed A, Ahmed RA, Al Sabaan FS (2016). Diabetes mellitus in Saudi Arabia: A review of the recent literature. Curr Diabetes Rev.

[11] Alneami YM, Coleman CL (2016). Risk factors for and barriers to control type-2 diabetes among Saudi population. Glob J Health Sci.

[12] Robert AA, Al Dawish MA (2020). The worrying trend of diabetes mellitus in Saudi Arabia: An urgent call to action. Curr Diabetes Rev.

[13] Alghamdi A (2017). Knowledge, attitude, and practice pattern among general health practitioners regarding diabetic retinopathy, Taif, kingdom of Saudi Arabia. Sau Jr Heal Sci.

[14] Meo S. A (2023). IDF: Diabetes reports 2000-2045. JPMA. The Journal of the Pakistan Medical Association.

[15] (2022). CDC: Your chance to prevent type 2 diabetes. https://www.cdc.gov/diabetes/basics/prediabetes.html.

[16] Al-Zahrani JM, Aldiab A, Aldossari KK (2019). Prevalence of prediabetes, diabetes and its predictors among females in Alkharj, Saudi Arabia: A cross-sectional study. Ann Glob Health.

[17] Alateeq MA, Aljohani M, Kinani SS (2020). The prediabetes outcome at national guard primary health care centers in Riyadh, Saudi Arabia: Retrospective chart review. Cureus.

[18] Ojo TK, Joshua OO, Ogedegbe OJ, Oluwole O, Ademidun A, Jesuyajolu D (2022). Role of intermittent fasting in the management of prediabetes and type 2 diabetes mellitus. Cureus.

[19] Jin J (2021). Screening for prediabetes and type 2 diabetes. JAMA.

[20] (2021, December 21). CDC: The surprising truth about prediabetes. https://www.cdc.gov/diabetes/library/features/truth-about-prediabetes.html.

[21] Wafi A, Aqeel A, Zogel B (2022). Adherence to physical activity recommendations in the adult population of Jazan region. Cureus.

[22] (2022). IDF: Diabetes atlas. https://diabetesatlas.org/atlas/eighth-edition/.

[23] Centers for Disease Control and Prevention, 2021 2021 (2022). CDC: About the prediabetes risk test. https://www.cdc.gov/prediabetes/takethetest/about-the-test.html.

[24] Silvanus V, Dhakal N, Pokhrel A (2019). Community based screening for diabetes and prediabetes using the Indian diabetes risk score among adults in a semi-urban area in Kathmandu, Nepal. Nepal Medical College Journal. Retrieved October 27.

[25] Nagalingam S, Sundaramoorthy K, Arumugam B (2017). Screening for diabetes using Indian diabetes risk score. Inte Jr.

[26] Mohammad A, Ziyab AH, Mohammad T (2021). Prevalence of prediabetes and undiagnosed diabetes among Kuwaiti adults: A cross-sectional study. Diabetes Metab Syndr Obes.

[27] Alfetni Alfetni, Abduljabbar Abduljabbar (2022). Physical activity prevalence and barriers among type 2 diabetic patients, Kudai and Al-hijra primary health care center, Makkah, Saudi Arabia. Medical Science.

[28] Al-Kaabi J, Al-Maskari F, Saadi H, Afandi B, Parkar H, Nagelkerke N (2009). Physical activity and reported barriers to activity among type 2 diabetic patients in the United arab emirates. Rev Diabet Stud.

[29] Do VV, Jancey J, Pham NM, Nguyen CT, Hoang MV, Lee AH (2019). Objectively measured physical activity of Vietnamese adults with type 2 diabetes: Opportunities to intervene. J Prev Med Public Health.

[30] Almarshad MI, Algonaiman R, Alharbi HF, Almujaydil MS, Barakat H (2022). Relationship between ultra-processed food consumption and risk of diabetes mellitus: A mini-review. Nutrients.

[31] Rauber F, Steele EM, Louzada ML, Millett C, Monteiro CA, Levy RB (2020). Ultra-processed food consumption and indicators of obesity in the United Kingdom population (2008-2016). PLoS One.

[32] AlFaris NA, Alshwaiyat NM, Alkhalidy H (2022). Sugar-sweetened beverages consumption in a multi-ethnic population of middle-aged men and association with sociodemographic variables and obesity. Front Nutr.

[33] Al-Hanawi MK, Ahmed MU, Alshareef N, Qattan AM, Pulok MH (2022). Determinants of sugar-sweetened beverage consumption among the Saudi Adults: Findings from a nationally representative survey. Front Nutr.

[34] Kolb H, Martin S (2017). Environmental/lifestyle factors in the pathogenesis and prevention of type 2 diabetes. BMC Med.

[35] Cosgrove MP, Sargeant LA, Caleyachetty R, Griffin SJ (2012). Work-related stress and Type 2 diabetes: systematic review and meta-analysis. Occup Med (Lond).

[36] Virtanen M, Ferrie JE, Tabak AG, Akbaraly TN, Vahtera J, Singh-Manoux A, Kivimäki M (2014). Psychological distress and incidence of type 2 diabetes in high-risk and low-risk populations: the Whitehall II Cohort Study. Diabetes Care.

[37] Wilding JP (2014). The importance of weight management in type 2 diabetes mellitus. Int J Clin Pract.

[38] Gilis-Januszewska A, Barengo NC, Lindström J (2018). Predictors of long term weight loss maintenance in patients at high risk of type 2 diabetes participating in a lifestyle intervention program in primary health care: The DE-PLAN study. PLoS One.

[39] Kontochristopoulou AM, Karatzi K, Karaglani E (2022). Sociodemographic, anthropometric, and lifestyle correlates of prediabetes and type 2 diabetes in europe: The Feel4Diabetes study. Nutr Metab Cardiovasc Dis.

